# Using a Gaussian Graphical Model to Explore Relationships Between Items and Variables in Environmental Psychology Research

**DOI:** 10.3389/fpsyg.2019.01050

**Published:** 2019-05-09

**Authors:** Nitin Bhushan, Florian Mohnert, Daniel Sloot, Lise Jans, Casper Albers, Linda Steg

**Affiliations:** ^1^Department of Psychometrics and Statistics, University of Groningen, Groningen, Netherlands; ^2^Faculty of Science, Informatics Institute, Universiteit van Amsterdam, Amsterdam, Netherlands; ^3^Department of Environmental Psychology, University of Groningen, Groningen, Netherlands

**Keywords:** graphical model, exploratory analyses, subgroup analysis, community energy initiatives, data visualization methods

## Abstract

Exploratory analyses are an important first step in psychological research, particularly in problem-based research where various variables are often included from multiple theoretical perspectives not studied together in combination before. Notably, exploratory analyses aim to give first insights into how items and variables included in a study relate to each other. Typically, exploratory analyses involve computing bivariate correlations between items and variables and presenting them in a table. While this is suitable for relatively small data sets, such tables can easily become overwhelming when datasets contain a broad set of variables from multiple theories. We propose the Gaussian graphical model as a novel exploratory analyses tool and present a systematic roadmap to apply this model to explore relationships between items and variables in environmental psychology research. We demonstrate the use and value of the Gaussian graphical model to study relationships between a broad set of items and variables that are expected to explain the effectiveness of community energy initiatives in promoting sustainable energy behaviors.

Exploratory data analyses are an important first step in scientific research (Chatfield, 1985; Behrens, 1997). Exploratory analyses provide a first understanding of the relationships between items and variables included in a study, which enables researchers to better understand the data before opting for more complicated and sophisticated analyses. Exploratory analyses are of particular relevance in so-called problem-oriented fields such as environmental psychology, where researchers often study how variables from different theories can help to explain a phenomenon to help solve a problem. Furthermore, applied psychologists may often work on large projects in which people from different (sub) disciplines collaborate in understanding climate-change related topics (or other complex challenges). Such problem-oriented approaches often aim to examine multiple research questions and test multiple hypotheses and theories, typically with questionnaire studies. This can result in large multivariate datasets. In such situations, researchers would profit from exploratory methods and analyses that help them get a “feel” for patterns in their dataset in an intuitive manner.

In such cases, exploratory analyses may involve three steps. First, relationships between items included in a study can be explored to get some initial insights into whether items that are assumed to measure the same underlying construct are indeed correlated. Second, after aggregating individual items into relevant scales, researchers can explore relationships between variables, as they would expect on the basis of theory. Third, in cases where the dataset comprises of multiple groups, exploratory analyses are helpful to examine similarities and differences in relationships between these variables across groups.

In this paper, we aim to introduce the Gaussian graphical model as a novel exploratory analysis tool for applied researchers that provides an easy to grasp overview of relationships between items and variables included in a study. Specifically, we propose a step-by-step approach toward using Gaussian graphical models in environmental psychology (see Figure 1). First, we will demonstrate how researchers can use this method to explore the structure underlying the questionnaire and examine whether items that aim to measure the same construct are indeed correlated. Second, we will illustrate how Gaussian graphical models can be used to visualize relationships between variables included in a large set, which can help researchers to get a first insight into strength of relationships between variables, and explore whether these are in line with theory (see the non-yellow regions in Figure 2). Moreover, Gaussian graphical models can reveal relationships between variables researchers did not anticipate or theorize about, such as relationships between variables derived from different theories that were not examined in combination before (see the yellow regions in Figure 2). This may help building new theories to be tested in future studies (Tukey, 1977; Chatfield, 1985). Third, we will show how Gaussian graphical models can be used to explore differences in relationships between the variables included in a dataset between sub-groups.

**Figure 1:**
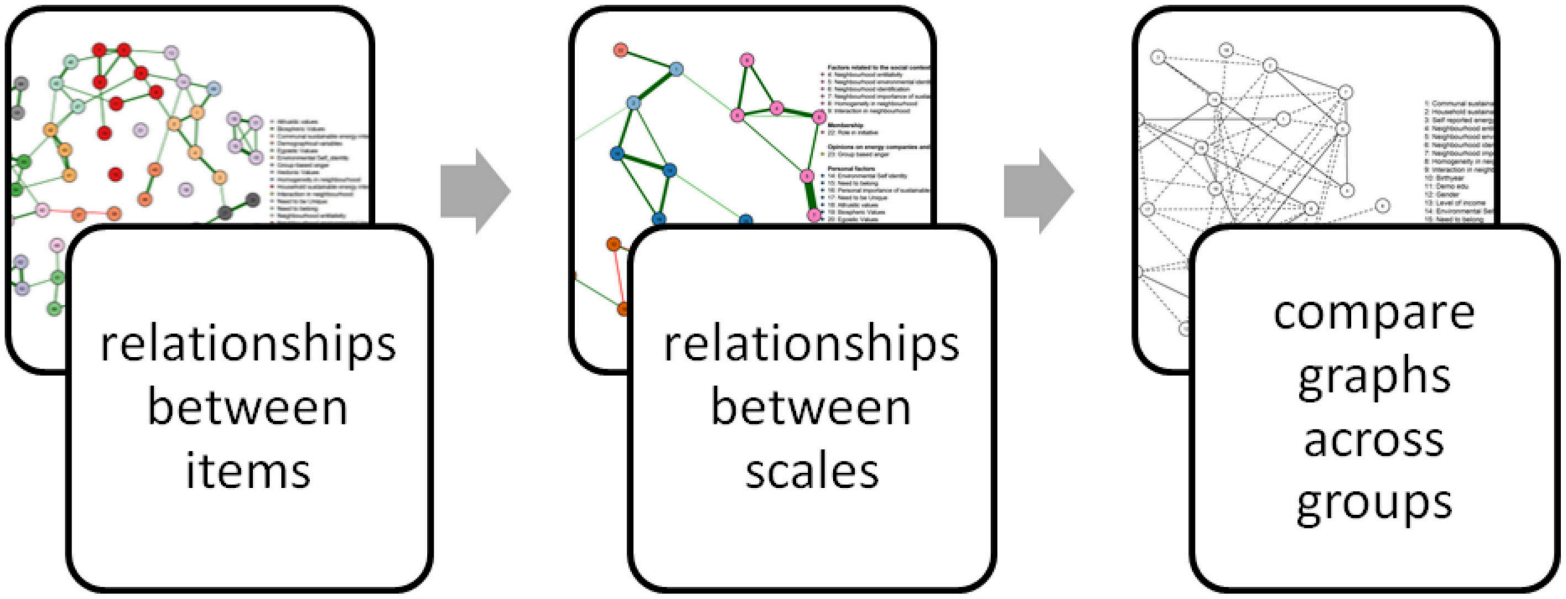
A systematic approach to exploratory data analysis using the Gaussian graphical model.

**Figure 2:**
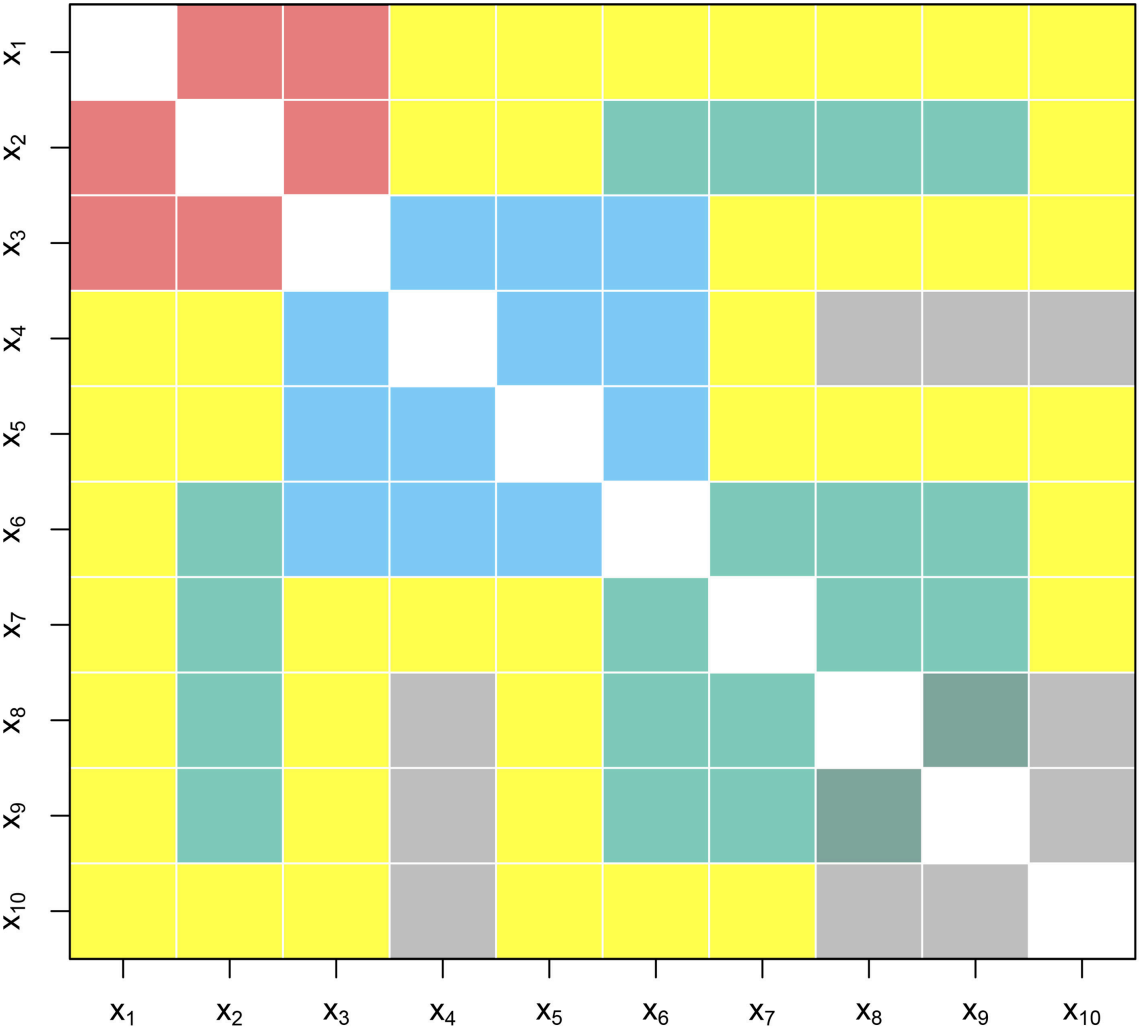
Combining theoretical perspectives can often result in new relationships previously not considered. Rows and columns denote variables. The squares denote relationships between variables. Here the non-yellow squares indicate relationships defined by theory. Yellow squares denote relationships that are yet to be discovered.

Gaussian graphical models have two advantages compared to common exploratory analysis that typically study bivariate correlations between items and variables. First, while bivariate correlations are useful in small datasets, correlational tables can become overwhelming in large datasets. Second, bivariate correlations between two variables can be spurious, i.e., caused by a third variable present in the dataset (a so-called common cause). In contrast, relationships estimated by Gaussian graphical models can be interpreted as partial correlation coefficients that reduce the risk of finding spurious relationships.

In addition, Gaussian graphical models have recently been applied in some fields in psychology, such as psychopathology and personality research (Cramer et al., 2012; Borsboom and Cramer, 2013) and it's technical origins can be traced back to Dempster (1972). However, to the best of our knowledge, these models have not been applied in problem-based fields such as environmental psychology, where they would have clear added value as stated above. Below, we briefly describe the Gaussian graphical modeling approach in an accessible manner and illustrate how it can be applied in environmental psychology research.

## 1. The Gaussian Graphical Model

A Gaussian graphical model comprises of a set of items or variables, depicted by circles, and a set of lines that visualize relationships between the items or variables (Lauritzen, 1996; Epskamp et al., 2018). The thickness of these lines represents the strength of the relationships between items or variables; and consequently, the absence of a line implies no or very weak relationships between the relevant items or variables. Notably, in the Gaussian graphical model, these lines capture partial correlations, that is, the correlation between two items or variables when controlling for all other items or variables included in the data set. As mentioned above, a key advantage of partial correlations is that it avoids spurious correlations.

**Figure 3:**
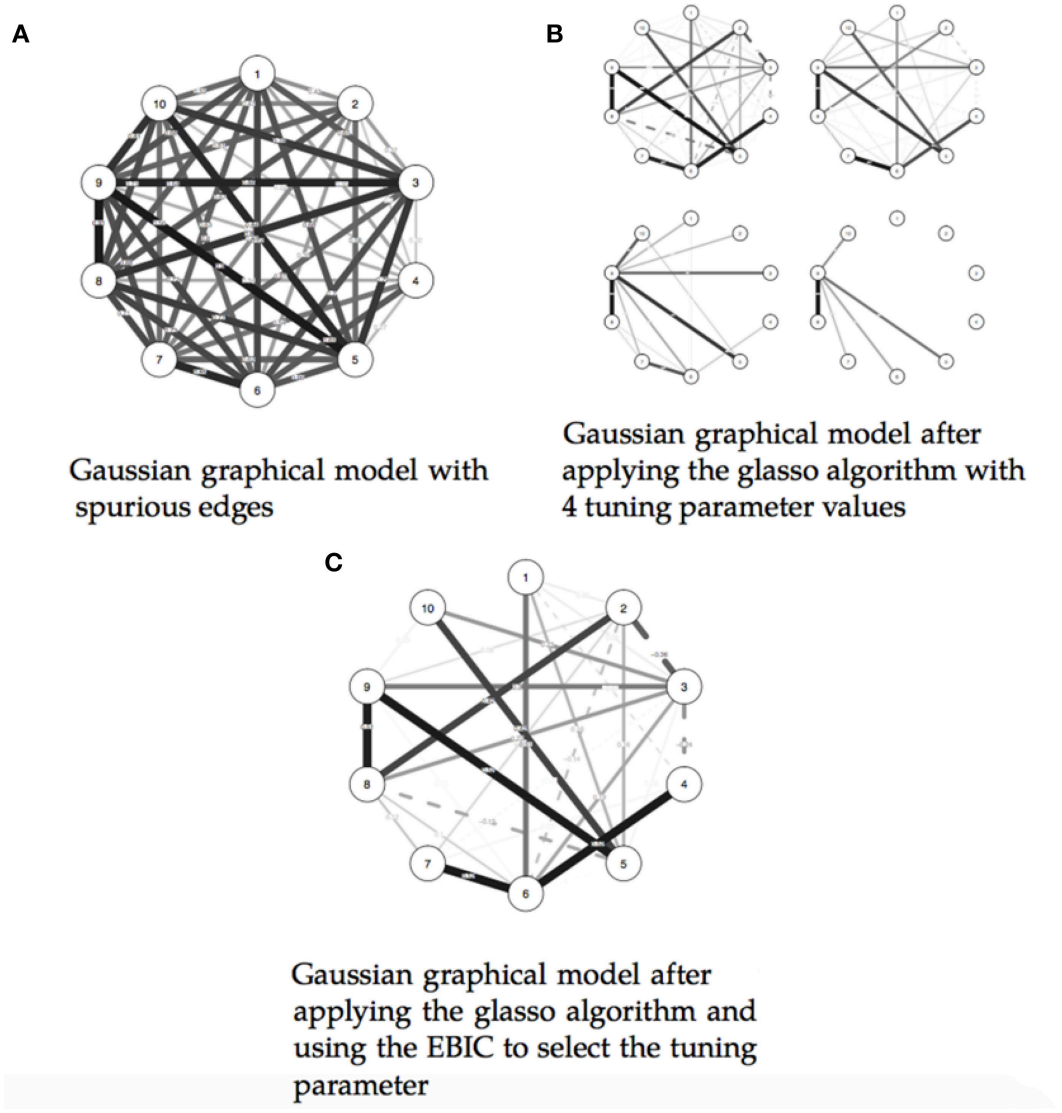
Illustrating the estimation of a Gaussian graphical model using the extended Bayesian information criteria (EBIC) and the glasso algorithm. Note that the EBIC optimally sets the turning parameters such that strong relationships are retained in the graph and weak relationships are set to zero. **(A)** Gaussian graphical model with spurious edges. **(B)** Gaussian graphical model after applying the glasso algorithm with 4 tuning parameter values. **(C)** Gaussian graphical model after applying the glasso algorithm and using the EBIC to select the tuning parameter.

While this visual representation of relationships can facilitate getting a first feel of the data, Gaussian graphical models can still be hard to read when the estimated graphs are dense and contain a large number of lines. In fact, due to sampling variation, truly zero partial correlations are rarely observed, and, as a consequence, graphs can be very dense and consist of spurious relationships (Epskamp et al., 2018). To this end, in Gaussian graphical models, the glasso algorithm is a commonly used method to obtain a sparser graph (Friedman et al., 2008). This algorithm forces small partial correlation coefficients to zero and thus induces sparsity. The amount of sparsity in the graph is controlled by a tuning parameter and different values of the tuning parameter result in different graphs (see Figure 3). Low values of the tuning parameter will result in dense graphs and high values of the tuning parameter will result in sparse graphs. Typically, the extended Bayesian information criteria (EBIC) is used to select an optimal setting of the tuning parameter (Foygel and Drton, 2010) such that the strongest relationships are retained in the graph (maximizes true positives). It is beyond the scope of this paper to describe the technical aspects of the Gaussian graphical model in detail, readers are guided to Epskamp et al. (2018) to understand the estimation of these models with a particular emphasis on their applications in psychology.

## 2. Similarity and Differences With Other Existing Models

The Gaussian graphical model is theoretically related to other exploratory modeling approaches in psychology, in particular with exploratory factor analysis to explore relationship between items included in a study. At the item level, there is indeed a similarity between the Gaussian graphical model and a uni-dimensional factor model (Lauritzen, 1996; Whittaker, 2009; Epskamp et al., 2018). A uni-dimensional factor model is a one factor model where the observed variables are independent conditional on the latent variable. This means that the correlations between items should tend do zero once we account for the latent variable. Consequently, a cluster of fully connected items indicates that these items measure a single latent construct. Hence, at the item level, this equivalence can be exploited to obtain insight into the factor structure of the questionnaire, which is recommended by methodologists (Schmitt, 1996; Crutzen and Peters, 2017).

The Gaussian graphical model differs from typical exploratory analysis based on partial correlational coefficients. Notably, a Gaussian graphical model shows relationships between items and variables in a graph, which is more easy to interpret than a large partial correlation table, particularly when small correlations are forced to zero via the glasso algorithm as we illustrate in the following application.

## 3. Application: Illustrating the Value of the Gaussian Graphical Model

We illustrate the use and value of the Gaussian graphical model for environmental psychologists and other applied researchers, by exploring relationships between items and variables included in a large dataset collected for a research project on community energy initiatives. This project aimed to study the psychological factors that can explain whether and why community energy initiatives may be effective in fostering sustainable energy behaviors (see Sloot et al., 2018). Specifically, community energy initiatives aim to promote sustainable energy behaviors in the communities in which they are established. Therefore, the researchers reasoned that social factors may play an important role in understanding the effectiveness of community energy initiatives, next to personal factors that have been shown to motivate sustainable energy behaviors (see Steg et al., 2015, for a review).

First, the researchers assumed that personal factors that have been shown to motivate sustainable energy behaviors may also predict sustainable energy behaviors in the context of community energy initiatives. Additionally, they assumed that these personal factors may motivate membership in these initiatives, as membership in a community energy initiative can be considered a specific type of sustainable energy behavior (Stern, 2000). Particularly, they considered the role of biospheric values as a general predictor of pro-environmental behavior (Steg et al., 2014), environmental self-identity as a more proximal predictor of sustainable energy behavior (van der Werff et al., 2013), and the personal importance people place in sustainable energy behavior in explaining sustainable energy behavior and pro-environmental behavior in general (see Sloot et al., 2018). Additionally, they assumed that these personal factors may motivate membership in these initiatives, as membership in a community energy initiative can be considered a specific type of sustainable energy behavior (Stern, 2000).

Second, they assumed that membership would motivate sustainable energy behaviors too (Sloot et al., 2018). Particularly, on the basis of the social identity approach (Turner, 1991; Tajfel and Turner, 2001), they theorized that groups we belong to, such as community energy initiatives, can form an important part of how we see ourselves (our social identity). When people think of themselves as members of a community energy initiative, they are likely to internalize the values and goals of this initiative and act accordingly, and collaborate with other members to further the group's goals. Given that community energy initiatives seem to have the explicit goal of promoting sustainable energy behaviors, membership to these groups may promote sustainable energy behaviors, and cooperation to achieve sustainable energy goals.

Third, they reasoned that a social identity lens may also help to better understand whether people will become a member in community energy initiatives (Sloot et al., 2017). While becoming an initiative member can be understood through personal factors that have been shown to motivate sustainable energy behaviors, it may also be influenced by the social context in which these groups are embedded. Particularly, the researchers reasoned that the communities in which community energy initiatives are embedded, can be regarded as groups, which can influence their members. Specifically, they considered the extent to which these communities could be seen as having a shared identity. They assumed that the more strongly inhabitants perceived their community as a strong entity, in terms of being a distinctive category and a dynamic entity (cf., Postmes et al., 2005; Jans et al., 2011), that places importance in sustainable energy behavior, and the more an individual identifies with this community (cf. Postmes et al., 2013), the more likely they would be to join an energy initiative in their community, and in turn engage in sustainable energy behaviors.

In addition, the social identity approach suggests that people are more likely to mobilize as a group, when there is a clear out-group they want to distance themselves from that elicits negative emotions (Van Zomeren et al., 2004; Postmes et al., 2006). In the context of community energy initiatives, particularly group-based anger and distrust about poor energy policies of the government and large energy companies may mobilize people to change the energy system by participating in community energy initiatives.

Next, the authors considered the need to belong and the need to be unique as two personal factors that motivate people to get involved in groups (cf. Brewer, 1991; Hornsey and Jetten, 2004), which may also motivate community energy membership.

In order to understand the relationships between personal factors, social factors, and the effectiveness of community energy initiatives, this project thus integrated variables from different theories. The questionnaire included different measures, of which some were newly created to fit the purpose of this particular study.

The above approach resulted in a very large dataset, for which exploratory analyses with the use of correlation tables would be hard to interpret (see Supplementary Materials). In such instances, the Gaussian graphical model can facilitate the researchers in their exploratory analyses in a systematic manner.

Below, we first demonstrate the use of the Gaussian graphical model to get a first insight into the relationships between the newly created items and other items included in the questionnaire. Second, we explore relationships between variables included in the study, and whether relationships were in line with what the researchers expected on the basis of their theorizing. This second step may also reveal relationships between personal factors and social factors that had not been anticipated by the researchers, which could stimulate theory development to be tested in future research. Third, we demonstrate the use of the Gaussian graphical model to examine whether relationships are similar for members and non-members of a community energy initiative.

### 3.1. Sample

A questionnaire study was conducted among members and non members of 29 community energy initiatives (varying in size) across the Netherlands that were part of an overarching network called Buurkracht (Buurkracht, 2018). In total of 568 participants completed the questionnaire. Of these, 303 reported to be members of the community energy initiative while the other 265 were non-members (see Sloot et al., 2018 for more details about data collection).

### 3.2. Measures

In this study, we included 32 variables reflecting the concepts introduced above, that were measured with 68 items. As indicated above, we included variables from personal factors, factors related to the social context, evaluations (or opinions) about energy companies and the government, self-reported sustainable energy behaviors and intentions to engage in sustainable energy behaviors (within the household and with the community) and other pro-environmental and communal behaviors, socio-demographical variables and membership of the community energy initiative. We elaborate on these measures below. Unless otherwise specified, items were measured on a 7-point Likert scale, ranging from 1 “completely disagree” to 7 “completely agree.”

#### 3.2.1. Personal Factors

##### 3.2.1.1. Values

Sixteen items measured the extent to which people endorse altruistic, biospheric, egoistic, and hedonic values (de Groot and Steg, 2008). Participants indicated how important each value is as a guiding principle in their life on a scale ranging from −1 (= against my principles) to 7 (= very important).

##### 3.2.1.2. Environmental self-identity

Three items measured the extent to which participants see themselves as an environmentally-friendly person (e.g., I am the type of person who acts environmentally friendly; van der Werff et al., 2013).

##### 3.2.1.3. Personal importance of sustainable energy behavior

Three items aimed to measure the extent to which participants find it important to engage in sustainable energy behavior (e.g., I find it important to be conscious about energy usage).

##### 3.2.1.4. Need to belong

One item measured the need to belong to a group. I find it important to belong to a group (adapted from Nichols and Webster, 2013).

##### 3.2.1.5. Need to be unique

One item measured the need to be unique. I find it important to be unique (adapted from Lynn and Snyder, 2002).

#### 3.2.2. Factors Related to the Social Context

##### 3.2.2.1. Neighborhood entitativity

We included one item to measure the extent to which the neighborhood was seen as an entity: In my opinion, the residents of my neighborhood are a coherent unit (Jans et al., 2011).

##### 3.2.2.2. Neighborhood homogeneity

Two items reflected to what extent people believe that people in their neighborhood have similar characteristics and thus can be seen as a clear category (e.g., Inhabitants of my neighborhood are similar to each other; Leach et al., 2008).

##### 3.2.2.3. Neighborhood interaction

Two items reflected the level of interaction among neighborhood inhabitants in general (e.g., Inhabitants of my neighborhood talk a lot with each other).

##### 3.2.2.4. Interaction with neighbors

Two items reflected the extent to which participants themselves interact with other inhabitants in their neighborhood (e.g., I speak a lot with other inhabitants of my neighborhood).

##### 3.2.2.5. Neighborhood identification

Four items measured to what extent participants identified with their neighborhood (e.g., I identify with my neighborhood; Postmes et al., 2013).

##### 3.2.2.6. Environmental neighborhood identity

Three item measure environmental neighborhood identity in a similar way as environmental self-identity (e.g., Inhabitants of my neighborhood are the type of people who act environmentally friendly).

##### 3.2.2.7. Neighborhood importance of sustainable energy behavior

The items reflecting personal importance of sustainable energy behavior were adapted to the level of the neighborhood. Hence, three items aimed to measure the extent to which participants think people in their neighborhood find it important to engage in sustainable energy behavior (e.g., Inhabitants of my neighborhood find it important to be conscious about energy usage).

#### 3.2.3. Evaluations of Energy Companies and the Government

##### 3.2.3.1. Group-based anger

Two items measured participant's anger toward energy policies by the government and large energy corporations, respectively (e.g., I am angry about the energy policies of the government [large energy companies]; adapted from Van Zomeren et al., 2004).

##### 3.2.3.2. Group-based distrust

Two items measured participant's distrust toward the government and large energy corporations, respectively (e.g., I have little confidence that the government [large energy companies] want to realize sustainable energy supply; adapted from Van Zomeren et al., 2004).

#### 3.2.4. Sustainable Energy Behavior and Intentions

##### 3.2.4.1. Sustainable energy behavior

Participants reported the extent to which they engage in sustainable energy behavior. One item captured overall energy savings (“To what extent did you reduce your energy consumption over the last 6 months?”) on a 7-point Likert scale, ranging from 1 (not at all) to 7 (very much). Three other items tapped into specific household energy behaviors. First, participants reported the current temperature setting (in °C) of their thermostat at home (open question). To achieve a distribution closer to normality the answers were trimmed to a range between 15 and 22°C. Second, they indicated their average showering time in minutes. Thirdly, participants indicated the percentage of energy efficient appliances in their household; scores could range from 0 to a 100%. Lastly, participants indicated for a range of investment measures (installing solar panels, double-glazing, roof insulation, floor insulation, wall insulation, and other) whether they did or did not intend to adopt each measure, or had already done so. Similar to Sloot et al. 2018, we counted the number of measures that participants reported to have adopted already; the resulting sum score could range between 1 (none of measures implemented) and 7 (all listed measures adopted).

##### 3.2.4.2. Household sustainable energy intentions

Five items aimed to measure participants' intention to engage in sustainable energy behavior in their household. Two reflect intentions to engage in sustainable energy behavior in general (i.e., lower your energy consumption; use more sustainable energy) while three reflect intentions to engage in specific energy saving behavior (i.e., set your thermostat lower, take shorter showers; replace household appliances with more energy efficient ones). Scores could range from 1 “not at all” to 7 “very much.”

##### 3.2.4.3. Communal sustainable energy intentions

Two items captured the extent to which participants intended to influence, and collaborate with, other community members to realize sustainable energy goals (e.g., to what extent do you intend to motivate others in your local community to save energy).

##### 3.2.4.4. Initiative involvement intentions

One item measured the intention of the participants to actively participate in their community energy initiative.

##### 3.2.4.5. Other pro-environmental intentions

Three items aimed to measure participants' intention to engage in other pro-environmental behaviors not directly targeted by the community energy initiatives (i.e., drive less; buy more pro-environmental products, donate money to a pro-environmental cause). All items were measured with a 7-point Likert scale ranging from “not at all” to “very much.”

##### 3.2.4.6. Other communal intentions

Two items tapped into intentions to engage in social activities with others in the neighborhood, unrelated to energy (e.g., To what extent do you intend to do fun things with other people in your community, not related to energy).

#### 3.2.5. Socio-Demographical Variables

Participants indicated their gender (binary; 1 indicates male), age, education level, and their level of household income.

#### 3.2.6. Membership

Participants indicated whether or not they are a member of their local community energy initiative. Participants could choose from 5 levels of membership ranging from not a member to the initiative taker. Higher levels of membership indicates greater involvement in the initiative.

### 3.3. Data Preparation and Analysis

The statistical software R version 3.4.1 (R Core Team, 2017) was used to apply the Gaussian graphical model on the dataset. We computed mean scores for items assumed to be belonging to the same scale to form variables. To estimate the Gaussian graphical model, we first need to estimate the correlation matrices at the item and scale level, respectively.

To handle missing data, we adopt a full information maximum likelihood (FIML) procedure using the *corFIML* function from the R package psych (Revelle, 2018). This procedure assumes that the data is multivariate normal. This assumption is not strictly met in our dataset and there is slight deviation from normality. However, FIML methods are robust to deviations from multivariate normality and studies have shown that they result in less biased estimates than *ad-hoc* approaches such as pairwise deletion (Enders, 2001; Enders and Bandalos, 2001; Schafer and Graham, 2002; Dong and Peng, 2013).

Next, using the estimated correlation matrices as input, the Gaussian graphical model was estimated using the *glasso* algorithm (Friedman et al., 2014). The graphs were then visualized using the R package qgraph (Epskamp et al., 2012). In qgraph, variables which are strongly correlated are placed spatially close to each other based on the Fruchterman Reingold algorithm (Epskamp et al., 2012), however, this does not imply that they are in anyway semantically or conceptually similar (for more details about this visualization algorithm, see Jones et al., 2018).

The key strength of the graphs is their ease of interpretation. The thickness of the line indicates the strength of the relationship. Next, green lines indicate positive partial correlation coefficients and red lines indicate negative partial correlations. For the sake of clarity, partial correlations with an absolute value below 0.1 are not visualized. Furthermore, for interested readers, the correlation matrices and the R script used to obtain the graphs are provided in the Supplementary Materials.

## 4. Results

### 4.1. Relationships Between Items Included in the Questionnaire

**Figure 4:**
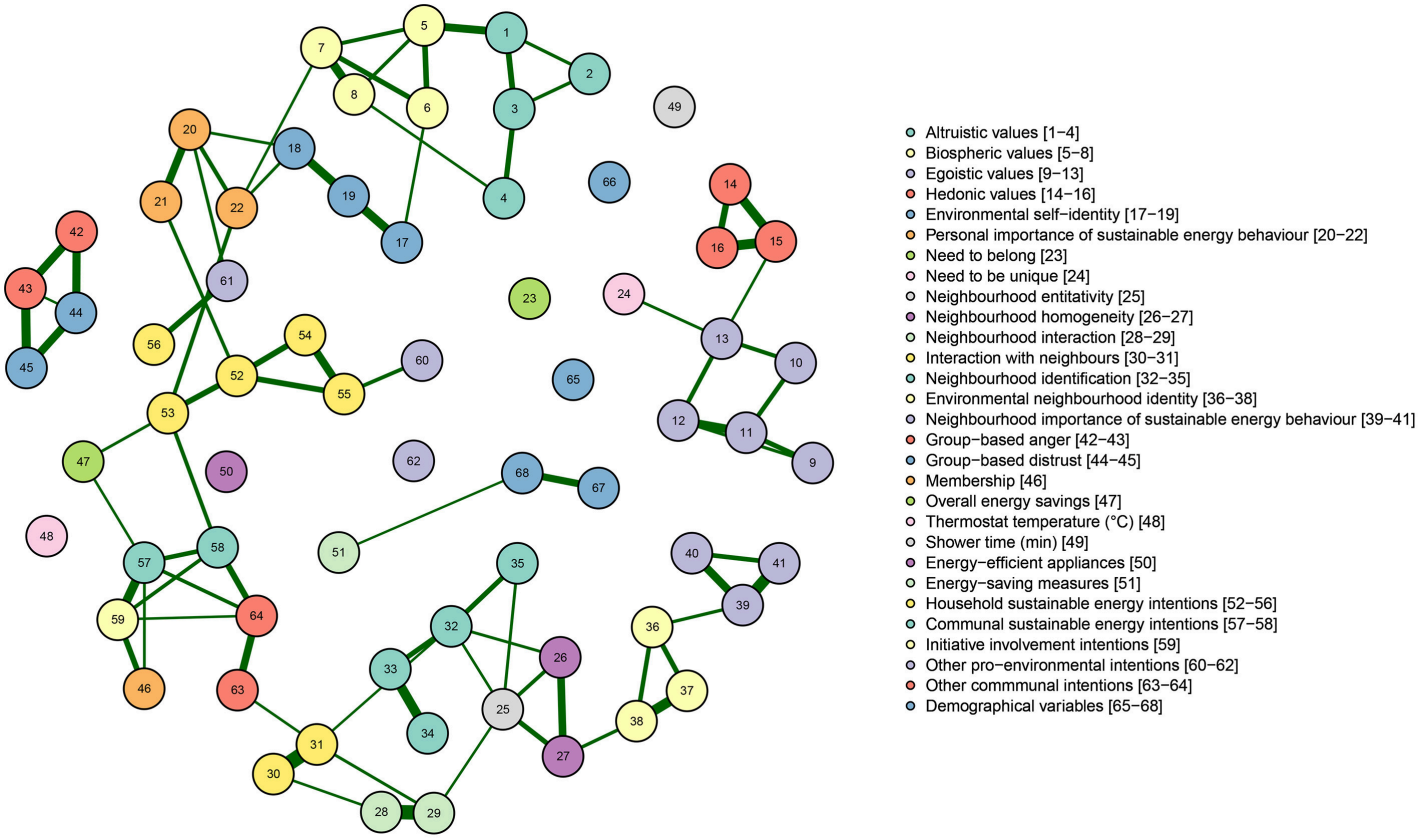
Gaussian graphical model displaying relationships between items. Items belonging to a scale are grouped by color. Note that items belonging to a scale tend to form clusters and items within a cluster exhibit stronger relationships than between clusters. Partial correlations with an absolute value below 0.1 are not displayed for sake of clarity.

Figure 4 displays the Gaussian graphical model representing relationships between items. Items that are densely connected with each other are called a cluster, indicated that the items are correlated, which provides a first insight into uni-dimensionality i.e., whether items that are supposed to measure the same variable are indeed related. We find that items included in a scale that measures a specific variable (depicted in the same color) are generally rather strongly related, and form clusters. For example, we can observe near perfect fully connected clusters of items measuring hedonic values, biospheric values, and sustainable energy intentions, respectively. In addition, Gaussian graphical model can also be used to examine inter-relatedness of items. Figure 4 indicates that relationships between items included in the same scale are generally more abundant than relationships between items assigned to different scales. Thus, by using a Gaussian graphical model, we get first insights into (i) whether items that are included in the same scale are indeed measuring the same thing (ii) whether items included in different scales are less strongly related, suggesting little overlap between the constructs included in the study.

### 4.2. Relationships Between Scales Included in the Questionnaire

**Figure 5:**
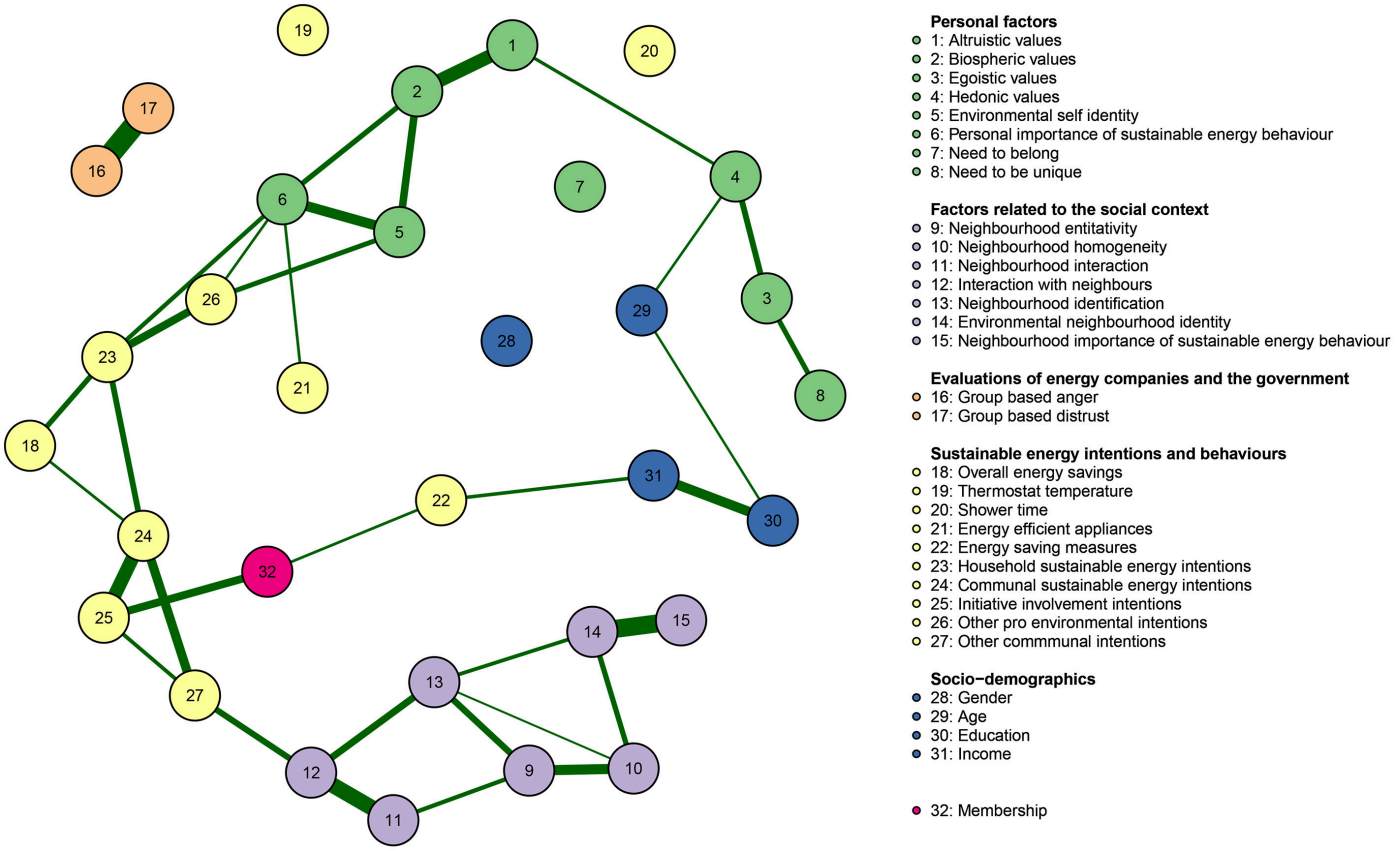
Gaussian graphical model displaying relationships between psychological constructs underlying community energy initiatives. Green lines indicate positive relationships and red lines indicate negative relationships. The color of the circle corresponds to the category the variable belongs to (e.g., biospheric values belong to personal factors). Partial correlations with an absolute value below 0.1 are not displayed for sake of clarity.

Next, we computed mean scores on items that were assumed to measure the same underlying variables and visualize the relationship between these variables using a Gaussian graphical model. Variables belonging to the same category (i.e., personal factors, factors related to the social context, evaluations of energy companies and the government, sustainable energy behaviors and intentions, and socio-demographics, and membership, respectively) are displayed in the same color. Similar to Sloot et al. (2018), we included all self-reported behavior items separately in the analysis. Figure 5 indicated relatively strong relationships (as indicated by the thickness of the lines) between the variables within constructs belonging to the same category (as indicated by the color of the circles).

First, we observe strong positive partial correlations between personal factors that are in line with common theorizing. For example, in Figure 5, biospheric values are positively related to environmental self-identity when controlling for the other variables (e.g., van der Werff et al. 2013). Furthermore, we see that that the more specific types of pro-environmental motivations, environmental self-identity and personal importance of sustainable energy behavior, are both related to pro-environmental intentions. Further, environmental self-identity is positively associated with household sustainable energy intentions via other pro-environmental intentions.

Similarly, as may be expected, the factors related to the social context were correlated. For example, in line with previous research, increased neighborhood identification was related to a stronger environmental neighborhood identity (cf. Masson et al., 2016).

Furthermore, we found a relationship between membership and initiative involvement intentions, while membership was only indirectly related to household sustainable energy intentions (i.e., via communal sustainable energy intentions) after controlling for the other variables. Furthermore, personal factors (green circles in the graph) form a chain that is linked with household sustainable energy intentions. These findings suggest that personal factors are more strongly related to household sustainable energy intentions, whereas initiative membership is more strongly related to communal sustainable energy intentions.

Interestingly, the Gaussian graphical model reveals that some variables seem not or hardly to be related to other variables included in the analysis. For example, while egoistic and hedonic values are strongly related, they exhibit weak or no relationships with sustainable energy intentions and behavior, which suggests that they do not mobilize nor inhibit people to act pro-environmentally in the context of community energy initiatives. Group-based anger and group-based distrust, while strongly related to each other, are hardly related to any of the variables, suggesting they are not very relevant to understand sustainable energy behaviors and intentions in the context of community energy initiatives. Furthermore, it is interesting to note that group-based anger does not relate to membership, which we might expect if as initiative involvement may be seen as a type of collective action (e.g., Van Zomeren et al., 2004; Bamberg et al., 2015).

### 4.3. Comparison of Relationships Between Variables Across Members and Non-members of Community Energy Initiatives

**Figure 6:**
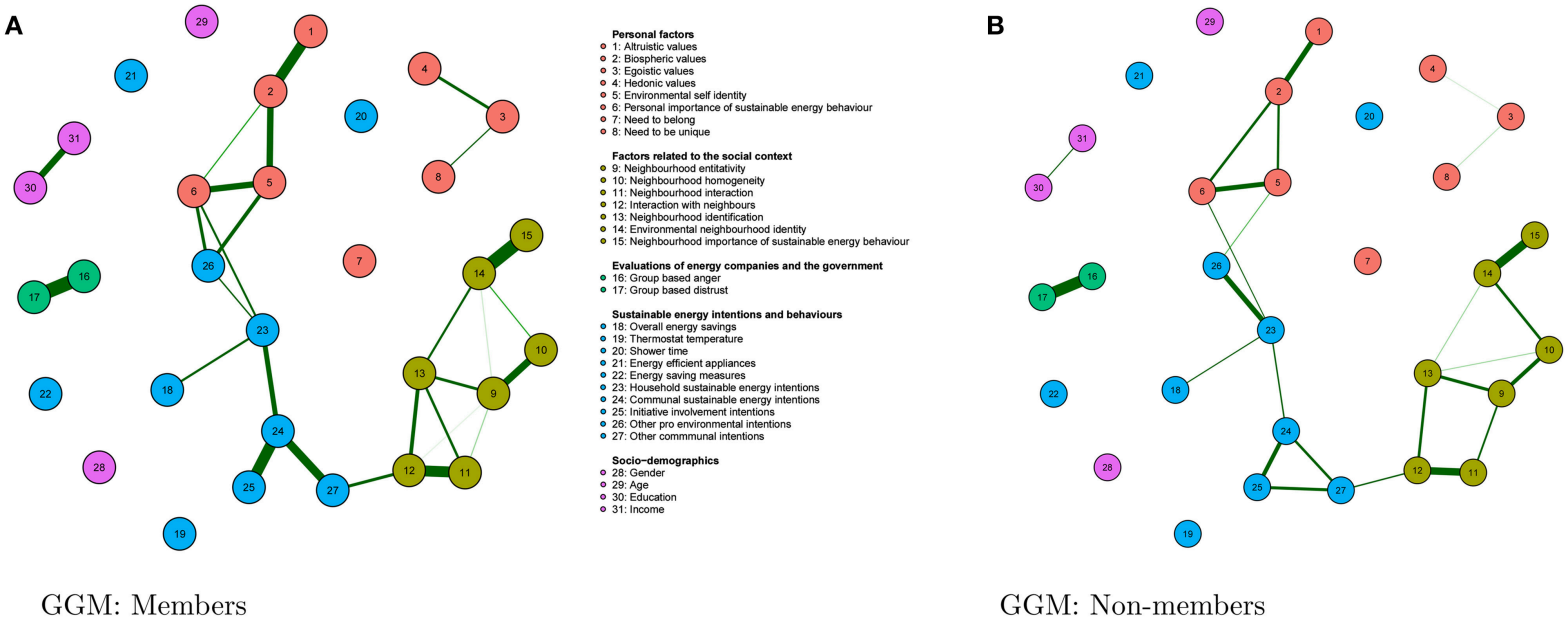
Graphs displaying relationships for members **(A)** and non-members **(B)**. Note that the overall patterns are similar across the graphs indicated by a structural hamming distance of 9. Partial correlations with an absolute value below 0.1 are not displayed for sake of clarity.

After looking at the relationships between variables, we lastly compared whether these relationships would differ between initiative members and non-members. Figure 6 reveals that the graphs are mostly similar for initiative members and non-members.

We quantify similarities (and differences) in the relationships between variables included in the graphs for the initiative members and non-members (see Figure 6) using the so-called structural Hamming distance. This measure only takes into account the presence (and absence) of relationships present in the graph and disregards the strength of the relationships. The smaller the structural Hamming distance, the greater the similarity between the graphs; a structural Hamming distance of zero indicates that the relationships between variables are identical.

In our case, the structural Hamming distance was 6, which implies that out of all estimated relationships between variables with an absolute value above 0.1 in members and non-members, approximately 98% (459 out of 465 possible relationships) of the relationships are similar across members and non-members of community energy initiatives. This suggests that strong relationships between the factors related to sustainable energy behaviors are very similar for members and non-members.

## 5. Discussion and Implications

We demonstrated the use of Gaussian graphical model to explore relationships between items and variables in large datasets aimed to understand the effects of community energy initiatives on sustainable energy behaviors and other type of pro-environmental and community behaviors. First, we found that the items belonging to a scale are strongly related, while partial correlations between items belonging to different scales were much lower, suggesting that there is little conceptual overlap between variables. Second, results suggest that most relationships observed are in line with theory. Furthermore, exploratory analysis using the Gaussian graphical model did not reveal unexpected relationships between personal factors and factors related to the social context i.e., the yellow regions of Figure 2 which could be the case when combining two theoretical perspectives not combined before. In addition, we found that relationships between variables were very similar for members and non-members of the community energy initiatives.

Our result suggest that the Gaussian graphical model is an useful tool to explore large datasets. Yet, a few points must be considered when using and interpreting results from this model. First, as these models capture partial correlation coefficients, all interpretations are conditional on the variables included in the model. To make the model and consequently, any interpretation meaningful, researchers must ensure that all variables relevant for the study are included.

**Figure A1:**
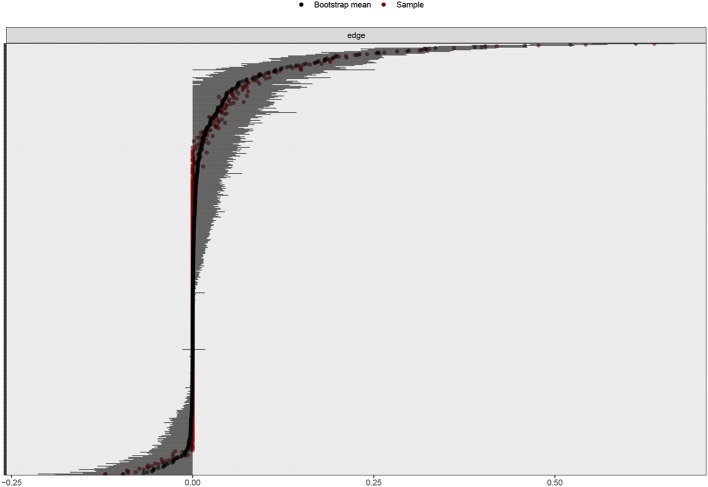
Gaussian graphical model stability analysis using the non-parametric bootstrap. The Y axis indicates the lines in the graph (omitted here for the sake of clarity). Red dots are the sample estimates and the black dots represent the bootstrap mean, i.e., the mean of all bootstrap replications obtained from re-sampling the original dataset (with replacement). The gray lines denote the bootstrap 95% confidence interval (CI) around the strength of a particular relationship. Note that in our case, the bootstrapped intervals of the strongest relationships in the network do not overlap the confidence intervals of the weakest edges. This indicates that the key relationships displayed in the graph are estimated reliably (Epskamp et al., 2018).

Second, as in any statistical model, researchers are advised to assess the stability of the results. One way of accessing the stability of the Gaussian graphical model is to use the so-called bootstrap method (Epskamp et al., 2018). This method accesses the stability of the model by generating several Gaussian graphical models based on re-sampled versions of the original dataset. The resulting models are then aggregated to obtain measures of accuracy and stability such as confidence intervals of the line weights (Epskamp et al., 2018). In our case, stability analysis using the non-parametric bootstrap revealed that the results are accurate in terms of estimated partial correlation coefficients (see Figure A1 in the Appendix). In particular, the bootstrapped intervals of the strongest relationships in the graph do not overlap the confidence intervals of the weakest relationships. This indicates that the key relationships displayed in the graph are estimated reliably (Epskamp et al., 2018).

Third, while comparing subgroups, we use the structural Hamming distance to quantify the similarity between graphs. It is important to note that this measure is descriptive and should not be interpreted as a formal statistical method to test for differences between graphs. In addition, the structural Hamming distance only compares graphs based on the presence and absence of lines and does not compare graphs based on the thickness of the lines (i.e., strength of partial correlation coefficients). This implies that two graphs which have similar relationships will appear to be strongly similar, even though the strength of the relationships may vary considerably between the two graphs. Rather, in explanatory research, this measure provides first insight into differences and similarities of variables between groups. Please note that there are methods to test for significant differences between graphs that also takes the thickness of the lines into account, but they often require strong assumptions about the distribution underlying the dataset in the population. For example, the *network comparison test* can be used for strictly multivariate normal or strictly binary datasets (van Borkulo et al., 2017) to test for statistically distinguishable differences in relationships between groups. In our application, the network comparison test indicated that the Gaussian graphical models for members and non-members do not statistically differ. However, we advise readers to use and interpret the results of this test with care. Firstly, the effects of non-normality on the network comparison test have not been investigated in detail. Secondly, using the network comparison test in the presence of unequal subgroup sizes and a penalized estimator such as the glasso increases the possibility of type-I errors, i.e., in reality, the differences between two graphs is much smaller than what we conclude on the basis of the test (van Borkulo et al., 2017).

Despite these limitations, the Gaussian graphical model can be a powerful tool to explore relationships between items and variables, particularly, when variables from multiple theories, not studied together are included in the model. It's key advantages include (i) an easy to understand visualization of relationships between items and variables, (ii) methods such as the glasso can be used to reliably estimate partial correlations that reduce the risk of finding spurious relationships, (iii) easy to use software (R and JASP), (iv) it is computationally fast, (v) the stability of the results can be accessed using the bootstrap method. Taking these advantages into account, we believe the Gaussian graphical model is a useful exploratory analysis tool which provides intuitive visualizations of key relationships for problem-based branches of psychology such as environmental psychology.

## 6. Conclusion

We present graphical models as a novel tool to explore relationships between items and variables in large datasets when researchers include variables from multiple theories (or disciplines) not studied together in combination before. Specifically, Gaussian graphical models facilitate researchers to get a first insight into (i) relationships between items included in their datasets, (ii) relationships between variables included in the dataset, and (iii) compare differences and similarities in relationships between variables included in the dataset between groups. Our results suggest that Gaussian graphical models can be particularly useful when researchers include variables from theories not studied together in combination before. In addition, these models can also be useful when experts from multiple (sub) disciplines collaborate in understanding climate-change related topics or other complex problems. Furthermore, this method not only provides some initial insights into relationships between items and variables, but can also lead to new theorizing, which can then be tested on a new dataset. Hence, Gaussian graphical models enable researchers to easily explore and understand relationships between large sets of variables that underlie the human dimension of the energy transition.

## Author Contributions

NB and FM conducted the analysis under the supervision of CA. NB, FM, LJ, and DS wrote a part of the paper and helped interpret the results. LS, LJ, and CA provided critical feedback on several versions of the manuscript. NB was the lead author, while all authors contributed to writing of the final manuscript.

### Conflict of Interest Statement

The authors declare that the research was conducted in the absence of any commercial or financial relationships that could be construed as a potential conflict of interest.
