# Supplementary material for: Using a Gaussian Graphical Model to Explore Relationships Between Items and Variables in Environmental Psychology Research
Source: Front Psychol. 2019 May 9;10:1050. doi: 10.3389/fpsyg.2019.01050 (PMC6521910; doi:10.3389/fpsyg.2019.01050)
Supplement: Supplementary file 1 [file Data_Sheet_1.ZIP › FrontiersGGMsupplementary/Images/itemGGM.pdf]

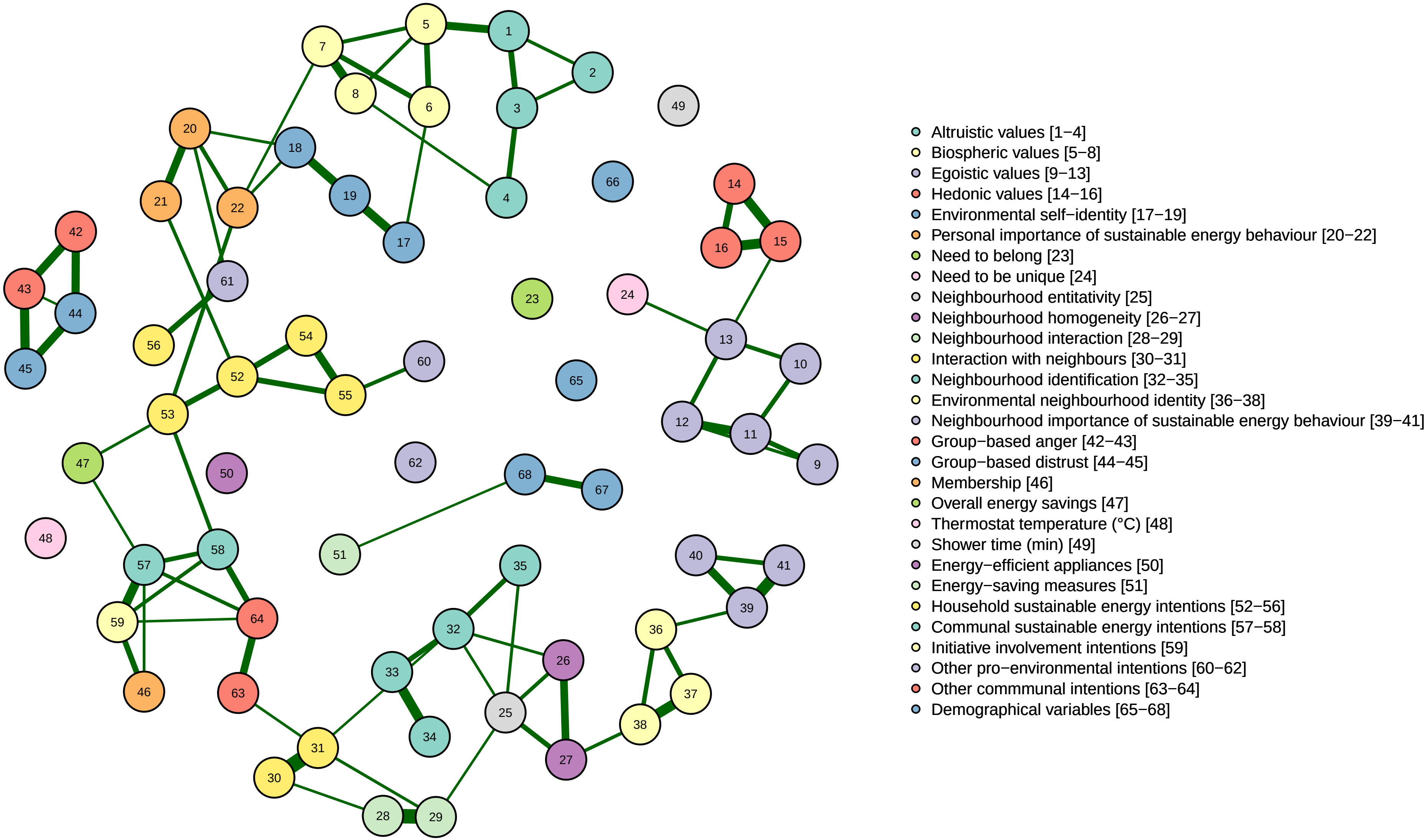
Altruistic values [1–4]
Biospheric values [5–8]
Egoistic values [9–13]
Hedonic values [14–16]
Environmental self–identity [17–19]
Personal importance of sustainable energy behaviour [20–22]
Need to belong [23]
Need to be unique [24]
Neighbourhood entitativity [25]
Neighbourhood homogeneity [26–27]
Neighbourhood interaction [28–29]
Interaction with neighbours [30–31]
Neighbourhood identification [32–35]
Environmental neighbourhood identity [36–38]
Neighbourhood importance of sustainable energy behaviour [39–41]
Group–based anger [42–43]
Group–based distrust [44–45]
Membership [46]
Overall energy savings [47]
Thermostat temperature (°C) [48]
Shower time (min) [49]
Energy–efficient appliances [50]
Energy–saving measures [51]
Household sustainable energy intentions [52–56]
Communal sustainable energy intentions [57–58]
Initiative involvement intentions [59]
Other pro–environmental intentions [60–62]
Other commmunal intentions [63–64]
Demographical variables [65–68]
