# Supplementary material for: Using a Gaussian Graphical Model to Explore Relationships Between Items and Variables in Environmental Psychology Research
Source: Front Psychol. 2019 May 9;10:1050. doi: 10.3389/fpsyg.2019.01050 (PMC6521910; doi:10.3389/fpsyg.2019.01050)
Supplement: Supplementary file 1 [file Data_Sheet_1.ZIP › FrontiersGGMsupplementary/Images/scaleGGM.pdf]

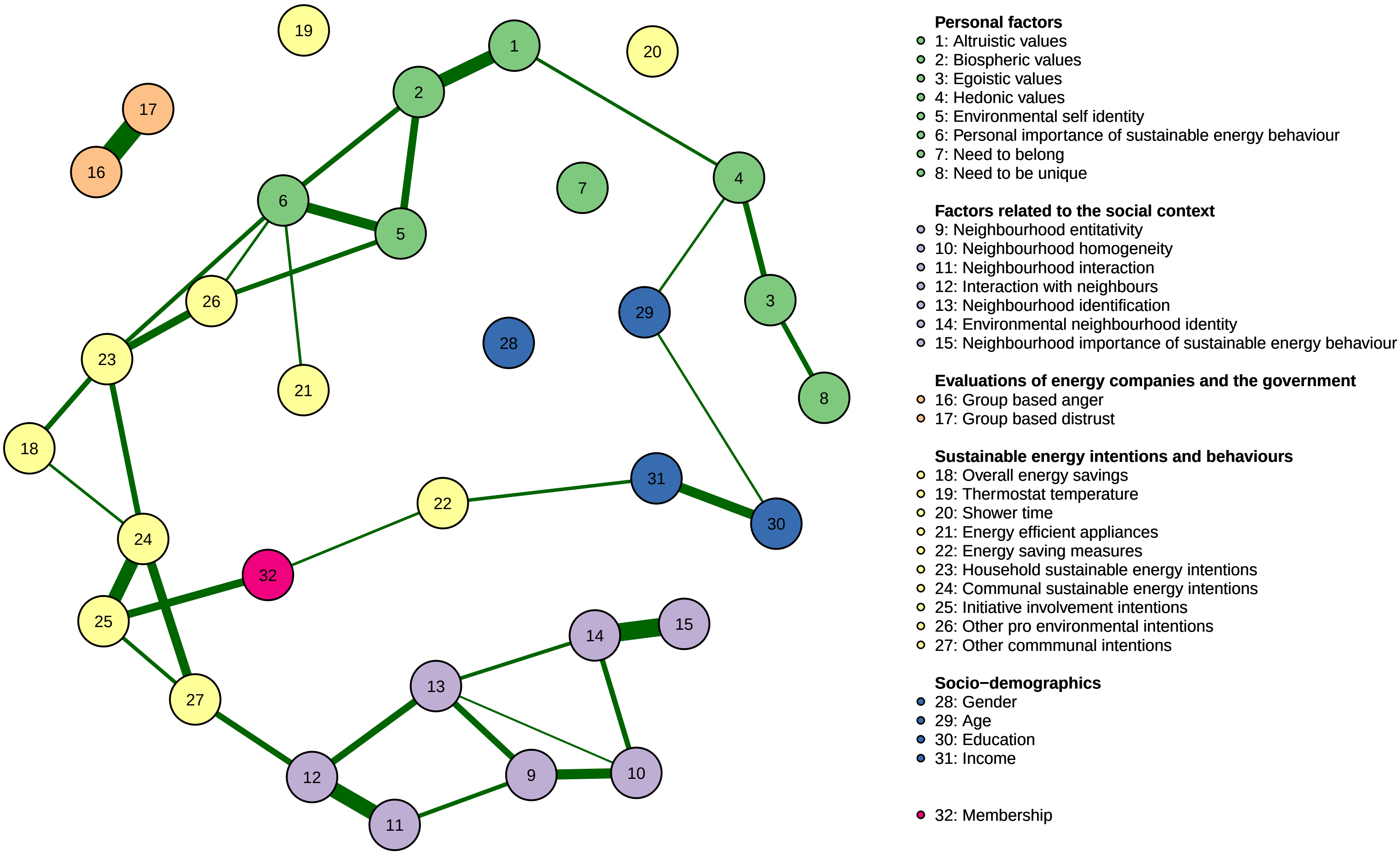

Personal factors
1: Altruistic values
2: Biospheric values
3: Egoistic values
4: Hedonic values
5: Environmental self identity
6: Personal importance of sustainable energy behaviour
7: Need to belong
8: Need to be unique
Factors related to the social context
9: Neighbourhood entitativity
10: Neighbourhood homogeneity
11: Neighbourhood interaction
12: Interaction with neighbours
13: Neighbourhood identification
14: Environmental neighbourhood identity
15: Neighbourhood importance of sustainable energy behaviour
Evaluations of energy companies and the government
16: Group based anger
17: Group based distrust
Sustainable energy intentions and behaviours
18: Overall energy savings
19: Thermostat temperature
20: Shower time
21: Energy efficient appliances
22: Energy saving measures
23: Household sustainable energy intentions
24: Communal sustainable energy intentions
25: Initiative involvement intentions
26: Other pro environmental intentions
27: Other commmunal intentions
Socio−demographics
28: Gender
29: Age
30: Education
31: Income
32: Membership
