# Supplementary figures and images for: Using a Gaussian Graphical Model to Explore Relationships Between Items and Variables in Environmental Psychology Research
Source: Front Psychol. 2019 May 9;10:1050. doi: 10.3389/fpsyg.2019.01050 (PMC6521910; doi:10.3389/fpsyg.2019.01050)

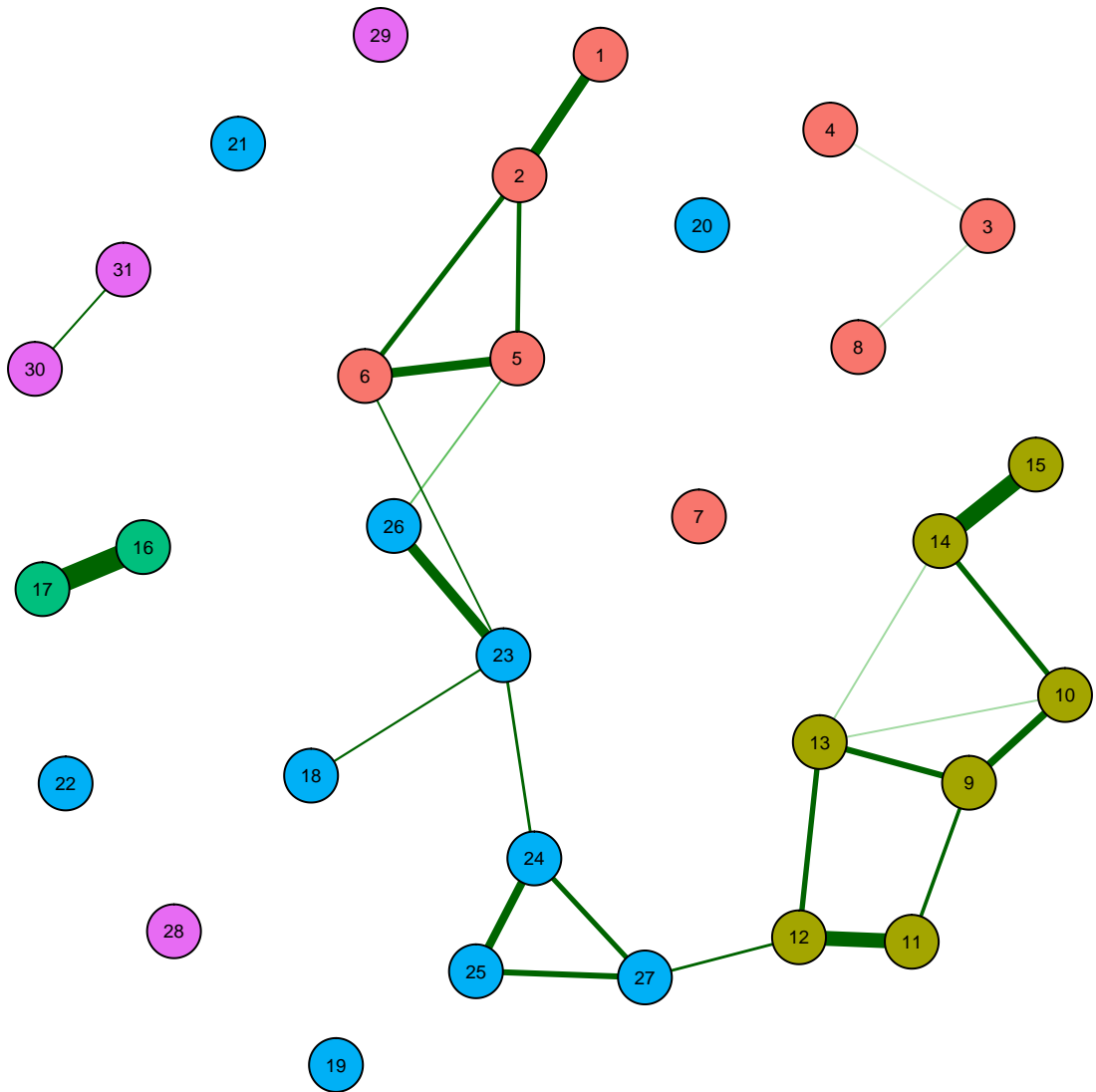

Supplement: Supplementary file 1 [file Data_Sheet_1.ZIP › FrontiersGGMsupplementary/Images/ScalesNonMember.pdf]
